# Fusion of the molecular adjuvant C3d to cleavage-independent native-like HIV-1 Env trimers improves the elicited antibody response

**DOI:** 10.3389/fimmu.2023.1180959

**Published:** 2023-05-22

**Authors:** Shridhar Bale, Lifei Yang, Mehrdad Alirezaei, Richard Wilson, Takayuki Ota, Esmeralda D. Doyle, Christopher A. Cottrell, Javier Guenaga, Karen Tran, Wenjuan Li, Leonidas Stamatatos, David Nemazee, Andrew B. Ward, Richard T. Wyatt

**Affiliations:** ^1^ Department of Immunology and Microbiology, The Scripps Research Institute, La Jolla, CA, United States; ^2^ International AIDS Vaccine Initiative, Neutralizing Antibody Center, The Scripps Research Institute, La Jolla, CA, United States; ^3^ Vaccines and Infectious Diseases Division, Fred Hutchinson Cancer Research Center, Seattle, WA, United States; ^4^ Department of Global Health, University of Washington, Seattle, WA, United States; ^5^ Department of Integrative Structural and Computational Biology, The Scripps Research Institute, La Jolla, CA, United States; ^6^ Consortium for HIV/AIDS Vaccine Development, The Scripps Research Institute, La Jolla, CA, United States

**Keywords:** HIV-1, C3d, immunogenicity, molecular adjuvant, vaccine

## Abstract

An effective HIV vaccine likely requires the elicitation of neutralizing antibodies (NAbs) against multiple HIV-1 clades. The recently developed cleavage-independent native flexibly linked (NFL) envelope (Env) trimers exhibit well-ordered conformation and elicit autologous tier 2 NAbs in multiple animal models. Here, we investigated whether the fusion of molecular adjuvant C3d to the Env trimers can improve B- cell germinal center (GC) formation and antibody responses. To generate Env-C3d trimers, we performed a glycine-serine- based (G_4_S) flexible peptide linker screening and identified a linker range that allowed native folding. A 30–60- amino- acid- long linker facilitates Env-to-C3d association and achieves the secretion of well-ordered trimers and the structural integrity and functional integrity of Env and C3d. The fusion of C3d did not dramatically affect the antigenicity of the Env trimers and enhanced the ability of the Env trimers to engage and activate B cells *in vitro*. In mice, the fusion of C3d enhanced germinal center formation, the magnitude of Env-specific binding antibodies, and the avidity of the antibodies in the presence of an adjuvant. The Sigma Adjuvant System (SAS) did not affect the trimer integrity *in vitro* but contributed to altered immunogenicity *in vivo*, resulting in increased tier 1 neutralization, likely by increased exposure of variable region 3 (V3). Taken together, the results indicate that the fusion of the molecular adjuvant, C3d, to the Env trimers improves antibody responses and could be useful for Env-based vaccines against HIV.

## Introduction

Despite many attempts both preclinically and in the clinic, a broadly effective vaccine preventing HIV-1 infection remains elusive. Such a vaccine likely requires the elicitation of cross-neutralizing antibodies (NAbs) directed toward the surface-exposed trimeric envelope glycoproteins (Env) (1). NAbs are the correlate of protection for most licensed vaccines and are a major focus of HIV-1 vaccine research efforts. In recent years, developments in soluble HIV trimer design has resulted in the generation of multiple soluble native-like mimics, including SOSIPs, NFLs, and UFOs that exhibit a well-ordered, native-like Env conformation (2–6). Antigenic evaluation of these trimers, and their more stabilized variants, demonstrates that they preferentially present broadly neutralizing antibody (bNAb) epitopes while occluding non-neutralizing determinants. Despite advances in design and improvement of the trimer mimics, the antibodies elicited by these recombinant glycoproteins are often limited to neutralizing “tier 1” (lab-adapted) viruses and autologous tier 2 viruses depending upon the viral strain from which the trimers derive or the animal model used (7–13). These data indicate that challenges remain to develop an effective HIV-1 vaccine (14–16). However, recently we and others have elicited antibodies with the capacity to neutralize a set of heterologous tier 2 clinical isolates in animal models, demonstrating a proof of principle that this is possible following sequential Env trimer immunization (17, 18).

**Figure 1:**
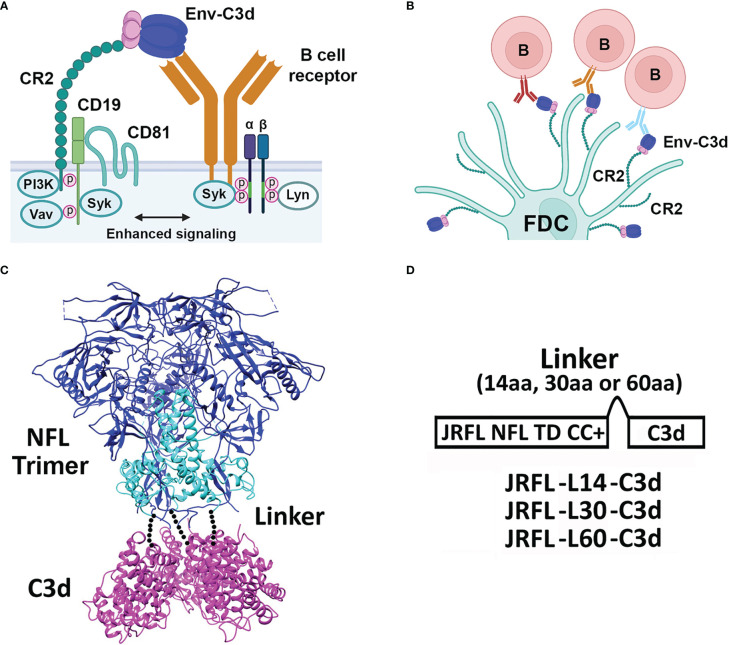
Activation by complement system, design, and schematic representation of HIV-1 Env-C3d fusion Trimers. **(A)** Cartoon model depicting enhanced activation of the innate immune system by simultaneous engagement of HIV-1 Env-C3d trimers with naïve B- cell receptors (BCRs) and CR2, CD19, and CD81 receptors of the complement system, respectively. **(B)** Further activation of the adaptive immune system in germinal centers by the engagement of CD2 receptors on follicular dendritic cells (FDCs) with BCR- attached Env-C3d trimers. **(C)** Model of Env-C3d (16055 NFL TD CC T569G, PDB 5UM8; human C3d, PDB: 3OED). The three Env gp120 subunits are shown in blue; the gp41 ectodomain subunits in cyan to form the gp140 trimers; mouse C3d in magenta. The three C3d subunits were manually fitted to the C-termini of the NFL trimer by visual inspection in PyMOL. The dotted lines represent the linker connecting the C-termini of NFL Env gp140 to the N-termini of C3d. **(D)** Linear schematic diagram of JRFL-C3d trimers. Mouse C3d was fused to the C-termini of NFL gp140 trimers by a flexible linker (G_4_S) of varying lengths (14, 30, and 60 amino acids to yield JRFL-L14-C3d, etc.). JRFL NFL TD CC+ (namely, JRFL) was used for the linker-length screening. The native signal peptide sequence of JRFL (or 426c NFL trimers) was replaced by the CD5 leader sequence to increase secretion in mammalian cells, as previously performed.

HIV-1 Env immunogens predominantly induce short-lived memory B- cell-dependent plasma Abs in the settings of both infection and vaccination (19). The magnitude and duration of antibody responses, affinity maturation, and the induction of B- cell memory are also important parameters to consider for development of an effective HIV-1 vaccine. Therefore, the means to improving the quality of the antibody responses is of great interest to the field and to basic B- cell biology, especially in the well-utilized mouse immune system. The use of molecular adjuvants, with specific and molecularly defined modes of action, has increased over the years (20). One relatively well-studied molecular adjuvant is a component of the complement pathway known as C3d (21). C3d is a cleavage product of complement component 3 and plays a key role in foreign antigen recognition, linking innate immunity to adaptive immune responses *via* binding to complement receptors (CD21, previously called CR2) (22). Following activation of the complement cascade by foreign proteins, C3d becomes covalently attached to the activating antigen. In turn, C3d can bind to CR2 present on B cells and follicular dendritic cells (FDCs), resulting in B- cell activation and initiation of an adaptive immune response (23) (**Figures 1A, B**, adapted from Carroll et al., Immunity 2012).

Dempsey et al. demonstrated previously that C3d can act as a molecular adjuvant when covalently fused to hen egg lysozyme (HEL), quantitatively lowering the amount of antigen needed to activate B cells 10,000- fold (21). We and others have used C3d fused to HIV-1 Env (gp120 or gp140) and confirmed the adjuvant effect of C3d in the absence of classical adjuvants but demonstrating less of an effect in the presence of a relatively robust “classical” adjuvant (24, 25). Very recently, it was shown that the lymph node follicles may be privileged sites with decreased proteolytic activity that may better maintain native antigen conformation (26). Thereby, targeting follicular FDCs may improve the elicitation of antibodies to native-like determinants. Accordingly, here we have sought to examine whether the fusion of C3d could improve the immunogenicity of the relatively recently developed conformationally near-native NFL Env trimers.

In this study, we genetically fused mouse C3d to the C-terminus of stabilized NFL Env trimers, previously developed and improved in our laboratory (4, 27). To accomplish this fusion, we initially performed a glycine-serine based (G_4_S) flexible peptide linker screening to identify a linker length that facilitated native-like trimer formation. We subsequently evaluated the Env-C3d fusion trimers regarding their biochemical, biophysical, and antigenic properties, as well as their conformational and functional integrity *in vitro*. Finally, we assessed the impact of C3d on the Env trimers regarding their immunogenicity *in vivo*, in the absence and presence of the Sigma Adjuvant System (SAS) adjuvant, in C57BL/6 mice and VRC01^gHL^ knock-in mice.

## Results

### Design and expression of HIV-1 Env-C3d fusion trimers

We fused mouse C3d to the well-ordered NFL gp140 Env to assess whether this immunostimulatory molecule can function as an effective adjuvant *in vivo*, to better target these native-like trimers to appropriate immune cells and more efficiently elicit antibody responses to these HIV vaccine candidates. We began this study by genetically fusing C3d sequences to the 3′-end of sequences encoding NFL gp140 trimers. The basics for the NFL trimer design are previously reported, along with stepwise improvements that included stabilizing “trimer- derived” (TD) mutations inferred from the crystal structure of the clade A BG505 SOSIP and trimer and inspection of the primary sequence of each stabilized Env trimer (JR-FL, 16055, etc.). The NFL construct also included a critical internal 201–433- cysteine bridging sheet linkage, now termed “CC1” that renders the trimer resistant to CD4-induced conformational changes (4, 5, 27). A cartoon representation depicting the details of the HIV-1 Env-C3d trimer design is shown in **Figure 1C**.

In this study, we began with the JR-FL NFL E160K TD CC+ trimer (“JRFL” in short for this work), as proof of principle to generate well-ordered Env trimers possessing genetically fused C3d from a single open reading frame. We covalently linked the N-terminus of C3d to the C-terminus of JRFL with a flexible linker (G_4_S) of varying lengths (**Figure 1D**). We determined previously that for cysteine linkage of the NFL trimers to maleimide-displaying liposomes, a cytoplasmic linker length greater than 13 residues is beneficial for efficient covalent linkage (28). Using this information, we first tested a 14- residue G_4_S linker (L14) between JRFL and C3d, designated as JRFL-L14-C3d. Following transient transfection in 293F cells, lectin purification, and size exclusion chromatography (SEC), we observed a broad protein peak instead of the expected distinct trimer peak in the SEC profile normally observed with the stabilized JR-FL trimers (5). Blue Native PAGE (BN-PAGE) revealed a broad protein “smear,” presumably non-native aggregates, with a small fraction of trimers (**Figure S1** **)**. The non-native conformation of the presumed aggregates was confirmed by antigenic profiling characterized by poor recognition by the trimer-specific broadly neutralizing antibody (bNAb), PGT145. Although this was an unexpected outcome, we reasoned that this was likely due to a suboptimal linker length to permit proper trimer folding when tethered to the C3d adduct. The overall globular structure of human C3d, as well as primary sequence, is highly conserved between humans and mice (29, 30). Accordingly, we increased the linker length to both 30 and 60 residues (designated as JRFL-L30-C3d and JRFL-L60-C3d, respectively) to potentially restore native-like trimer folding.

### The long linkers facilitate Env-C3d folding to achieve homogenous well-ordered, native-like conformations

**Figure 2:**
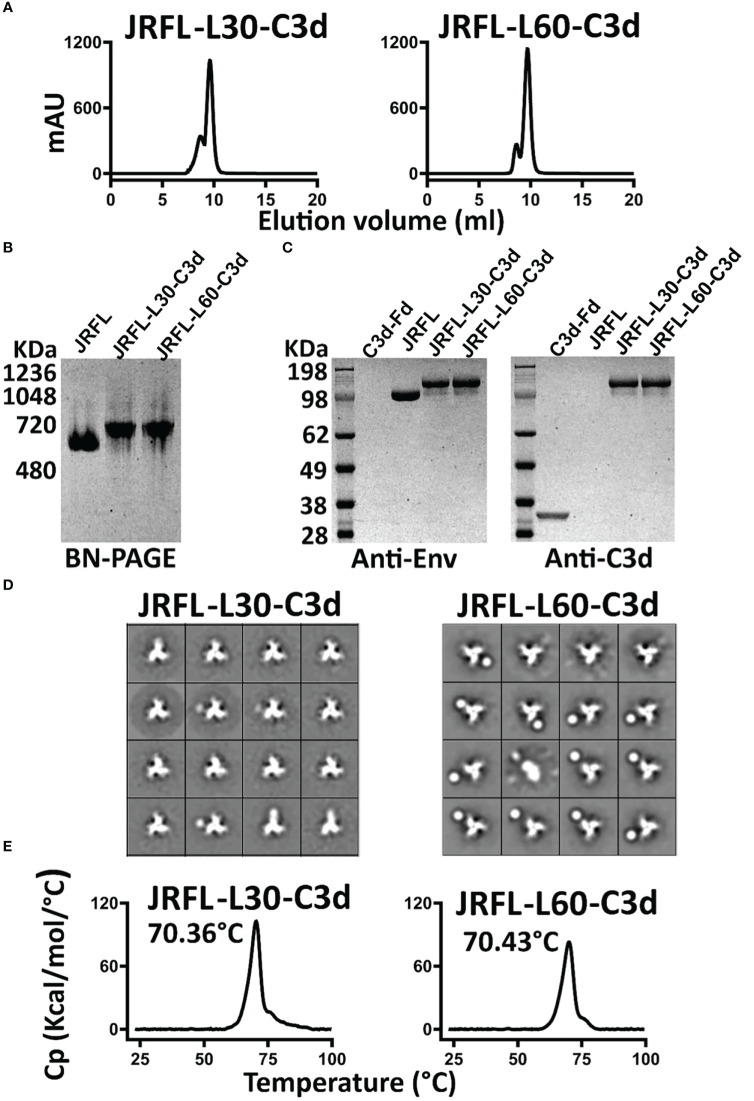
Biochemical and biophysical characterization of JRFL native-like-C3d fusion trimers. **(A)** SEC profile of JRFL-C3d trimers following lectin affinity purification and F105 negative selection. **(B)** Blue-native PAGE (BN-PAGE) analysis of JRFL-C3d trimers. **(C)** Western blot analysis of JRFL-C3d trimers. Protein identification was determined by anti-Env antibodies (2G12 and VRC01) or mouse C3d-specific antibody. Purified JRFL NFL native-like trimers and mouse C3d-foldon proteins were used as controls. **(D)** 2D averages from NS- EM of JRFL-C3d trimers revealed a native-like well-ordered structure. **(E)** DSC measurements of JRFL-C3d trimers. The T_m_ values are shown on top of each temperature peak.

The JRFL-L30-C3d and JRFL-L60-C3d variants were expressed in 293F cells and purified by lectin affinity purification followed by F105 negative-selection chromatography, as previously described (31). The two JRFL-C3d variants possessing the longer linkers appeared to form well-ordered trimers, each resolving as a single trimer peak by SEC (**Figure 2A**). For comparison, the SEC profile of the JRFL trimer is shown in **Figure S1C**. The yield of JRFL-L60-C3d trimers was approximately 0.8 mg/L, higher than that achieved by the JRFL-L30-C3d trimers (0.4 mg/L). The purified fusion proteins were resolved on BN-PAGE, revealing a migration pattern consistent with trimeric Env that generated a single band ~720 kDa by PAGE analysis (**Figure 2B**). The JRFL-L30-C3d and JRFL-L60-C3d fusions both migrated slightly more slowly in the gel than their counterpart JRFL trimers, consistent with the fusion of C3d adding ~90 kDa additional mass to the trimer (C3d is ~30 kDa). The successful fusion of C3d to JRFL was confirmed by Western blot analysis using Env-specific antibodies (2G12 and VRC01) and a mouse C3d-specific antibody, respectively (**Figure 2C**). Homogeneous trimer formation of JRFL-L30-C3d and JRFL-L60-C3d was also confirmed by negative stain-EM (NS-EM). NS-EM 2-D average analysis showed that JRFL-L30-C3d and JRFL-L60-C3d trimers were in closed, native-like conformation (**Figure 2D**). Then, we assessed the thermostability of JRFL-L30-C3d and JRFL-L60-C3d trimers by differential scanning calorimetry (DSC). JRFL-L30-C3d and JRFL-L60-C3d displayed a single thermal transition curve, confirming the homogeneity of trimers. The T_m_ of JRFL-L30-C3d and JRFL-L60-C3d were 70.38°C and 70.43°C, respectively (**Figure 2E**), values comparable with the T_m_ of JRFL (70°C) as previously described (27). These data indicated that the fusion of C3d to JRFL Env did not alter the thermostability or homogeneity of the trimer component.

### The Env-C3d fusion trimers exhibit similar antigenic profiles as their counterpart Env trimers

**Figure 3:**
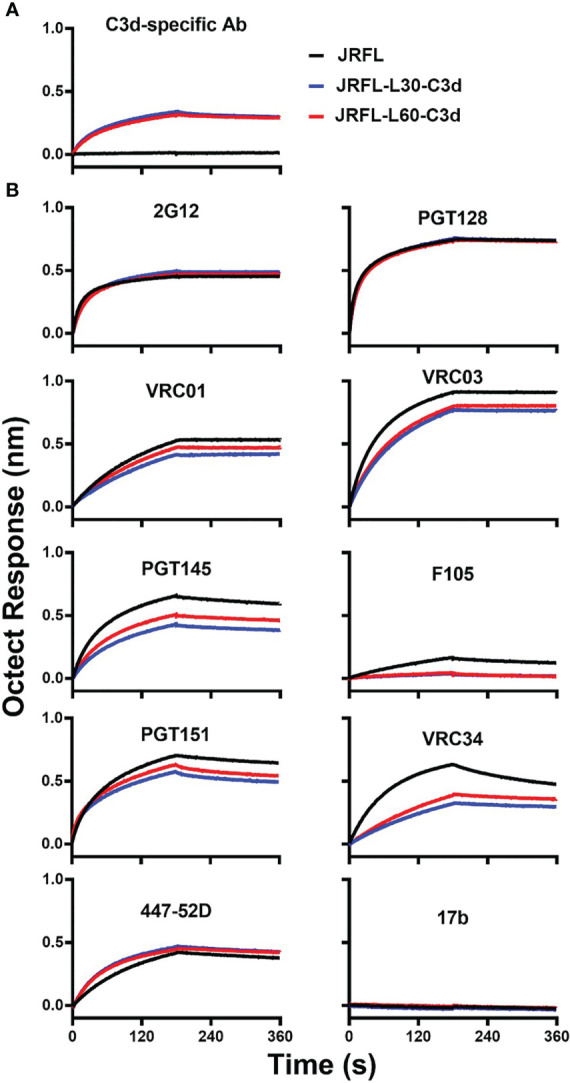
The JRFL-C3d trimers display a favorable antigenic profile as determined by Bio-layer interferometry (BLI). **(A)** JRFL-C3d trimers bind to the mouse C3d-specific antibody, which was immobilized on streptavidin sensors. Binding was not detected for JRFL trimer lacking the C3d domain. **(B)** JRFL and JRFL-C3d trimers are recognized by bNAbs and are not recognized by non-NAbs. Data are representative of at least two independent experiments.

To assess the potential impact of adding C3d to the JRFL Env trimers, we used a panel of mAbs to evaluate their level of binding to Env-C3d trimers and isogenic non-conjugated Env trimers. First, we tested recognition of JRFL-C3d trimers by C3d-specific Ab by Biolayer Interferometry. JRFL-L30-C3d and JRFL-L60-C3d showed similar recognition by the C3d-specific Ab, whereas there was no binding of their counterpart JRFL trimers, as expected (**Figure 3A** **)**. Next, we tested recognition of the JRFL-C3d trimers by Env-specific mAbs. The JRFL-L30-C3d and JRFL-L60-C3d fusions were recognized similarly compared with unmodified JRFL trimers by bNAbs targeting the N-glycan shield (2G12), the glycan-dependent V3 332N region (PGT128), and slightly lower binding to bNAbs targeting the CD4 binding site (VRC01, VRC03), V2-apex (PGT145), and fusion peptide (VRC34) (**Figure 3B** **)**. As expected, there was little or no binding detected by the non-neutralizing Abs F105 and 17b (**Figure 3B** **)**. Taken together, Env-C3d fusion trimers with L30 and L60 linkers exhibit similar antigenicity profiles. These data demonstrate that the fusion of C3d to the NFL Env trimers did not largely affect the antigenicity of the Env trimers but that the longer linker appeared to be more favorable for the Env-C3d trimers to maintain functional integrity.

### The fusion of C3d improves the ability of the Env trimers to engage and activate B cells *in vitro*

**Figure 4:**
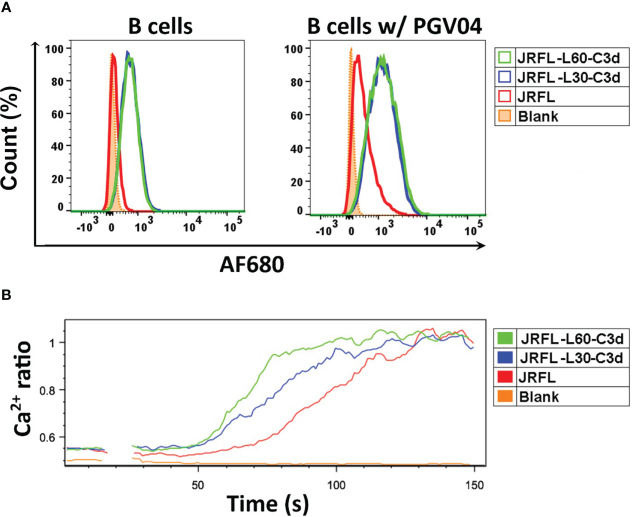
JRFL-C3d trimers bind and activate mouse B cells *in vitro*. **(A)** Flowcytometry analysis of JRFL-C3d trimers binding to mouse B cells with or without PGV04 expression on their surface. Percentage of maximum cell count is shown on the Y-axis, and relative fluorescence of Alexa Flour 680 (AF680) dye- conjugated trimers is shown on the X-axis. **(B)** Calcium flux analysis of B- cell activation by JRFL-C3d trimers. Mouse B cells expressing PGV04 on their surface were used. The resulting Ca^2+^ flux was measured over a period of 120 s. Representative data from two independent experiments is shown. JRFL trimers served as control for both the assays.

To evaluate whether the fusion of C3d increases the ability of Env trimers to engage mouse B cells through C3d and CR2 interaction, we labeled JRFL and fusion JRFL-C3d trimers with Alexa Fluor 680 dye (AF680) and performed binding analysis by flow cytometry. The results show that JRFL-L30-C3d and JRFL-L60-C3d trimers bound well to PBMC-derived mouse B cells presumably through a trimer-C3d to CR2 interaction on the B cells. As expected, there was no detectable binding of JRFL trimers to B cells because there are no Env-specific B- cell receptors (BCRs) expressed on these normal mouse B cells (**Figure 4A**).

JRFL trimers showed modest binding reactivity to PBMC-derived normal mouse B cells, which display CR2 (CD21) and CD4bs-directed bNAb, PGV04, on their surface. However, the binding reactivities of JRFL-L30-C3d and JRFL-L60-C3d trimers were substantially increased with the addition of PGV04 expression, indicating that C3d enhanced the engagement of JRFL-C3d trimers to the mouse B cells through both C3d–CR2 interaction and the Env-specific BCRs (**Figure 4A**).

To further evaluate whether the fusion of C3d enhances the capability of the Env trimers to activate mouse B cells *in vitro*, we performed calcium flux analysis of B cells expressing the bNAb PGV04 using a flow cytometric assay (28, 32). B cells were stimulated with 50 μg/ml of soluble JRFL or JRFL-C3d trimers. The resulting Ca^2+^ flux (measurement of a 405/485-nm emission ratio of Indo-1 fluorescence upon excitation by UV) was analyzed over 120 s. Under the conditions tested, Ca^2+^ flux responses followed the trend JRFL-L60-C3d > JRFL-L30-C3d > JRFL NFL (**Figure 4B**). The results indicated that JRFL-C3d trimers activated the B cells more efficiently than the JRFL trimers by cross-linking CR2 and the BCRs (C3d-CR2 and Env-PGV04 in this case). In addition, JRFL-L60-C3d trimers generated much stronger B- cell activation than JRFL-L30-C3d, suggesting that the longer linker is more favorable for JRFL-C3d fusion trimers to achieve C3d-mediated functional integrity by engaging CR2s present on the B- cell plasma membrane. Finally, JRFL-L60-C3d trimers showed strong binding to soluble human CR2 protein by biolayer interferometry (BLI) (**Figure S2**) that was not detected using JRFL trimers lacking C3d fusion.

Taken together, the fusion of C3d improved the ability of Env trimers to engage and activate B cells *in vitro*. The fusion Env-C3d trimers preserved the functional integrity of both Env and C3d to engage Env-specific BCR and CR2, to activate B cells. The longer linker L60 was more favorable for Env-C3d fusion trimers to preserve the functional integrity of both the Env trimers and C3d. Accordingly, we selected the Env-L60-C3d trimers for subsequent immunogenicity studies *in vivo*.

### Germinal center B cells are efficiently activated by JRFL-L60-C3d trimers

**Figure 5:**
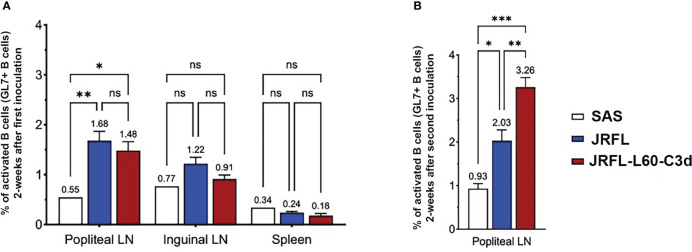
B cells from germinal centers in mice are better activated by JRFL-C3d trimers compared with those lacking C3d. **(A)** Comparison of activated GL7^+^ B cells measured by FACS after immunization with adjuvant- only, JRFL NFL, and JRFL-C3d trimers. Percent of activated GL7^+^ B cells were measured in draining popliteal and inguinal LNs and spleen 2 weeks after priming immunization. **(B)** Percent of activated GL7^+^ B cells in popliteal LN 6 weeks after two immunizations. The second immunization was performed at week 4. ns indicates non-significant. *, indicate *P* ≤ 0.05; **, indicate *P* ≤ 0.01; ***, indicate *P* ≤0.001.

We next tested the ability of JRFL-L60-C3d trimers to activate the germinal centers *in vivo* compared with the JRFL NFL trimer. Three groups of mice were immunized with JRFL NFL (13 mice), JRFL-L60-C3d (13 mice), and adjuvant SAS only (5 mice); 2 weeks after the immunization, six mice from the Env- immunized group and two mice from the adjuvant SAS only group were analyzed for GL7^+^ B cells (a surface marker specific for mouse germinal center B cells) in popliteal and inguinal lymph nodes (LNs) and spleens by flow cytometry. The fraction of GL7^+^ B cells in the population of CD45^+^/CD19^+^ B cells was found to be popliteal LNs > inguinal LNs > spleen. Although there was a trend, there was no statistically significant difference in the fraction of activated GL7^+^ B cells between the groups of mice immunized with JRFL or JRFL-L60-C3d trimers 2 weeks post priming immunization **(** **Figure 5A** **)**. The remaining mice were boosted at week 4 and analyzed at week 6 for GL7^+^ B cells in the draining popliteal LNs as they had the highest signal. Activated GL7^+^ B cells in the mice immunized with JRFL-L60-C3d trimers were higher than those of mice immunized with JRFL NFL trimers (p = 0.0032) as well as for the adjuvant- only group (p = 0.0001) **(** **Figure 5B** **)**.

### Env-C3d induces stronger anti-Env binding antibody responses

**Figure 6:**
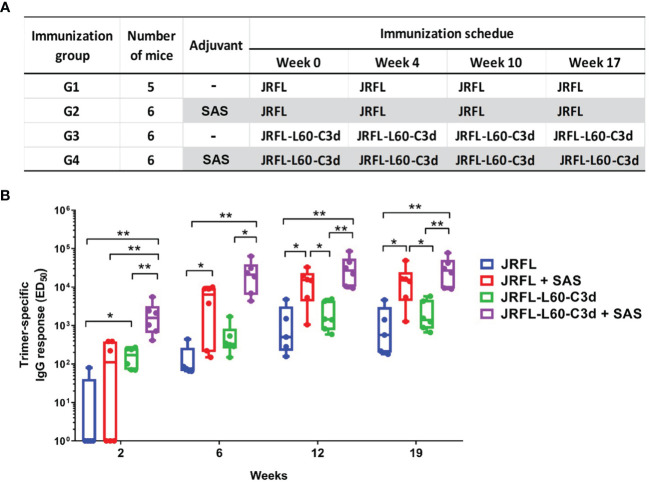
Immunization schedule and comparison of Env-specific binding antibody responses elicited in mice. **(A)** Immunization groups, antigens, and vaccine regimen. **(B)** JRFL trimer-specific IgG-binding titers were determined using 2G12-capture ELISA. JRFL-C3d fusion trimers elicit statistically significant higher binding antibodies than trimers lacking the C3d domain. The ED_50_ of each animal plotted, with geometric mean and min–max values shown. The box plots illustrate the following: horizontal line, median; plus, mean; box, interquartile range; whiskers, min/max. Statistical differences were evaluated by two-tailed Mann–Whitney U test. * indicates *P* ≤ 0.05; ** indicates *P* ≤ 0.01.

Next, we assessed whether the fusion of C3d can improve antibody responses elicited by the near-native HIV-1 Env NFL trimers *in vivo*. As outlined in **Figure 6A**, four groups of C57BL/6 (B6) mice were immunized with JRFL or JRFL-L60-C3d trimers in PBS (no adjuvant) or SAS adjuvant, which has supplanted the RIBI adjuvant system. This adjuvant contains MPLA and other immune-stimulating compounds in an emulsion formed by squalene, Tween 80, and water. Following the first immunization, all the animals in the JRFL-L60-C3d- immunized groups generated Env-specific antibodies; however, only one (in G1) and three (in G2) animals in JRFL- immunized groups generated Env-specific antibodies. Following the subsequent immunizations, all animals generated anti-Env antibodies and the magnitude of Env- binding antibodies increased significantly after the second immunization and plateau following the third and fourth immunizations (**Figure 6B**). The results showed that in the presence or absence of the SAS adjuvant, JRFL-L60-C3d trimers elicited higher-titer Env-specific IgG antibodies than JRFL (G3 vs. G1, G4 vs. G2), especially following the first immunization.

As shown in **Figure 6B**, the SAS adjuvant also enhanced the antibody responses for JRFL and JRFL-L60-C3d trimers in mice (G2 vs. G1, G4 vs. G3). Since adjuvant SAS contains MPLA, which is a TLR-4 agonist, we reasoned that the enhanced immunogenicity might be observed if both CD21 and TLR-4 pathways were targeted by the JRFL-C3d proteins in the presence of SAS. Indeed, the JRFL-C3d with SAS elicited significantly higher-titer Env-specific IgG antibodies than the JRFL without SAS after each immunization (G4 vs. G1). These data demonstrate that there is an additive effect of molecular adjuvant C3d and the SAS adjuvant to improve the immunogenicity of the Env trimers.

Overall, the results showed that molecular adjuvant C3d improves the ability of the Env trimers to elicit antibodies, to rapidly engage and activate naïve or germline B cells. In addition, there was an additive effect of C3d and the SAS adjuvant to improve the immunogenicity of the Env trimers.

### The fusion of C3d increases the avidity of Env-specific antibodies in the presence of adjuvant

**Figure 7:**
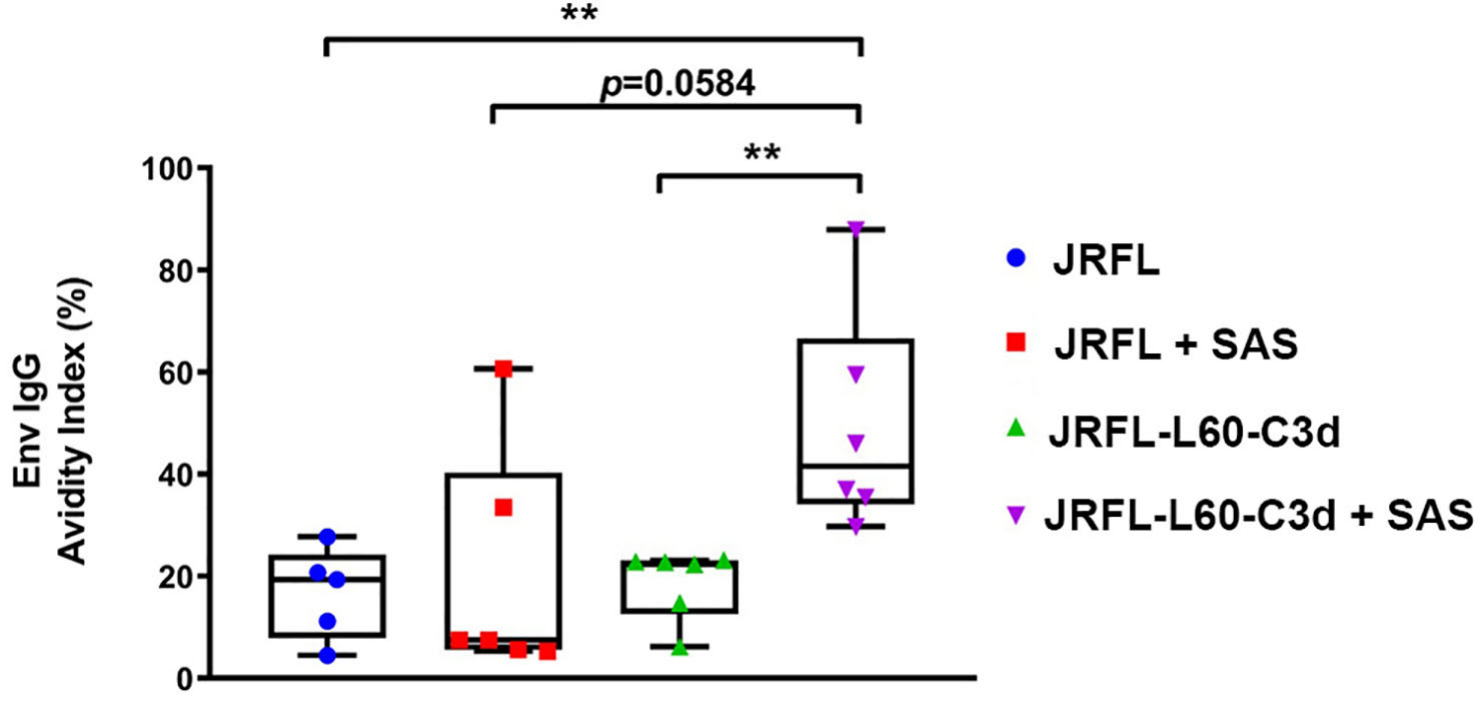
Avidity of the anti-Env IgG in mice after four immunizations. JRFL-specific NaSCN-displacement ELISA was used to measure the avidity index of anti-Env IgG in mouse sera at week 19 (2 weeks after the fourth immunization). The avidity index was calculated as the percentage of endpoint titers for NaSCN-treated samples to those of PBS-treated samples. The box plots illustrate the following: horizontal line, median; plus, mean; box, interquartile range; whiskers, min/max. Statistical differences were evaluated by two-tailed Mann–Whitney U test. * indicates P ≤ 0.05; ** indicates P ≤ 0.01. Data shown are representative from two independent experiments. JRFL-C3d trimers result in a statistically significant higher avidity index in the SAS adjuvant than in JRFL trimers alone in PBS and SAS adjuvant.

To investigate if fusion of C3d can improve the avidity of Env-specific antibody responses, we performed sodium thiocyanate (NaSCN) displacement ELISAs. The avidity of Env-specific antibodies after the final immunization was measured by using the chaotropic agent NaSCN to potentially disrupt lower- affinity Env-antibody interactions. Under the stringent binding assay involving incubation in 1.5 M NaSCN, the avidity of the antibodies elicited by SAS- adjuvanted JRFL-L60-C3d trimers was higher and trending toward significance than that from SAS- adjuvanted JRFL trimer- vaccinated mice (G4 vs. G2, p = 0.0584) (**Figure 7**). However, in the absence of the SAS adjuvant, the JRFL-L60-C3d trimers did not drive antibody avidity compared with their counterpart JRFL trimers. Taken together, these results indicated that the fusion of C3d to Env may increase the affinity maturation of Env-specific antibodies in mice in the presence of the SAS adjuvant.

### Env-C3d fusion trimers induce stronger tier 1 neutralizing antibody responses in the presence of adjuvant

The serum samples (with ED_50_ values shown in **Figure 6B**) were further assessed for the capacity to neutralize pseudo-typed viruses by the standard TZM-bl assay (33). As shown in **Table 1**, substantial HIV-1- specific NAbs were elicited after the third immunization against tier 1 virus MN.3 in animals immunized with trimers in the SAS adjuvant. Following the fourth immunization, the neutralizing antibody response against tier 1 virus MN.3 was further increased. No reliably detected neutralization was observed against the antigen-matched tier 2 JRFL virus as expected in a mouse model, as seen previously (28, 34). Surprisingly, in the absence of the SAS adjuvant, the JRFL and JRFL-L60-C3d trimers did not elicit NAbs against MN.3 virus. MN.3 is a tier 1 clade B virus, which is particularly sensitive to V3-directed antibodies, whereas JRFL, from which our Env immunogens are derived, is a relatively resistant tier 2 clade B virus. The inability of the JRFL and JRFL-L60-C3d trimers to elicit NAbs against MN.3 likely reflects the maintenance of the native-like and desired closed native trimer conformation in aqueous solution *in vitro*. Low- neutralization titers against tier-1 viruses were also observed in rabbits and non-human primates after immunizations with native-like NFL trimers (10, 13, 35). However, when the immunogens were formulated in the SAS adjuvant, the JRFL and JRFL-L60-C3d trimers elicited a relatively strong neutralizing antibody response against the tier 1 MN.3 virus, indicating that the SAS adjuvant may be partially disruptive to the trimer integrity *in vivo*, resulting in an increased exposure of the V3 element. This protein determinant is often immunogenic and leads to the elicitation of antibodies that can neutralize lab-adapted isolates such as MN.3.

**Table 1:** Neutralization ID_50_ titers for sera from immunized mice against a panel of pseudo-typed viruses.

		Week 0 Pre-Bleed			Week 6 Post 2			Week 12 Post 3			Week 19 Post 4			
JRFL	Animal #	SIV	MN.3	JRFL	SIV	MN.3	JRFL	SIV	MN.3	JRFL	SIV	MN.3	JRFL
	ZZ6148	<20	<20	<20	<20	<20	<20	<20	<20	<20	<20	<20	<20
	ZZ6149	<20	<20	<20	<20	<20	<20	<20	<20	<20	<20	<20	<20
	ZZ6150	<20	<20	<20	<20	<20	<20	<20	<20	<20	<20	<20	<20
	ZZ6151	<20	<20	<20	<20	<20	<20	<20	<20	<20	<20	<20	<20
	ZZ6152	<20	<20	<20	<20	<20	<20	<20	<20	<20	<20	<20	<20
JRFL SAS	ZZ6153	<20	<20	<20	<20	<20	<20	<20	<20	<20	<20	4995	<20
	ZZ6154	<20	<20	<20	<20	<20	<20	<20	<20	<20	<20	<20	<20
	ZZ6156	<20	<20	<20	<20	<20	<20	<20	<20	<20	<20	905	<20
	ZZ6157	<20	<20	<20	<20	<20	<20	<20	<20	<20	<20	<20	<20
	ZZ6158	<20	<20	<20	<20	2703	<20	<20	4790	<20	<20	4860	<20
	ZZ6159	<20	<20	<20	<20	<20	<20	<20	133	<20	<20	2051	<20
JRFL-L60-C3d	ZZ6160	<20	<20	<20	<20	<20	<20	<20	<20	<20	<20	<20	<20
	ZZ6161	<20	<20	<20	<20	<20	<20	<20	<20	<20	<20	<20	<20
	ZZ6162	<20	<20	<20	<20	<20	<20	<20	<20	<20	<20	<20	<20
	ZZ6163	<20	<20	<20	<20	<20	<20	<20	<20	<20	<20	<20	<20
	ZZ6164	<20	<20	<20	<20	<20	<20	<20	<20	<20	<20	<20	<20
	ZZ6165	<20	<20	<20	<20	<20	<20	<20	<20	<20	<20	<20	<20
JRFL-L60-C3d SAS	ZZ6166	<20	<20	<20	<20	<20	<20	<20	<20	<20	<20	65	<20
	ZZ6167	<20	<20	<20	<20	<20	<20	<20	210	<20	<20	8810	<20
	ZZ6168	<20	<20	<20	<20	<20	<20	<20	<20	<20	<20	<20	<20
	ZZ6169	<20	<20	<20	<20	<20	<20	<20	<20	<20	<20	341	<20
	ZZ6170	<20	<20	<20	<20	<20	<20	<20	<20	<20	<20	4860	<20
	ZZ6171	<20	<20	<20	<20	<20	<20	<20	7803	<20	<20	52387	<20

Data are representative of two independent experiments.

The boxes are colored as follows: none, ID_50_ < 20 = no neutralization; green, ID_50_ 20–100 = weak neutralization; yellow, ID_50_ 101–1,000 = moderate neutralization; brown/red, ID_50_ > 1,000 = strong neutralization. SIVmac239 (SIV in short), was used as negative control.

### The SAS adjuvant affects the immunogenicity of Env trimers *in vivo* but not their antigenicity *in vitro*

As described above, there is an additive effect of the molecular adjuvant C3d and the SAS adjuvant to improve the immunogenicity of the Env trimers. Interestingly, in the B6 mice immunization study, NAbs against MN.3 virus were only induced by immunogens in the presence of the SAS adjuvant, regardless of which antigen was inoculated into the mice. Neutralization against MN.3 is usually taken to indicate the elicitation of V3-directed antibodies to this “tier 1 virus” which exists in a more open Env spike conformation. As shown in **Figure S3A**, the SAS- adjuvanted immunogens elicited higher V3-directed antibodies than immunogens in PBS. It implies that the adjuvant SAS affects the trimer integrity *in vivo*. Moreover, there was a strong correlation of V3-specific binding antibody titers and the MN.3 neutralization titers (**Figure S3B**).

To further investigate whether the SAS adjuvant affects the Env trimer integrity *in vitro*, we tested the effect of SAS on the antigenicity of the Env trimers recognized by selected antibodies. We incubated His-tagged JRFL trimers in the presence or absence of the SAS adjuvant at room temperature or 37°C for 24 h. We captured the trimers on an ELISA plate *via* the His-tag and tested recognition of the trimers by a panel of mAbs. As shown in **Figure S4**, binding of the JRFL trimer to bNAbs or non-NAbs was quite similar in both the conditions tested, indicating that the SAS adjuvant did not affect the integrity of the trimer *in vitro*. Taken together, the SAS adjuvant likely alters the integrity of the JRFL trimers *in vivo*, as is evident by the elicitation of V3-directed binding and neutralizing antibodies even though SAS does not appreciably affect Env trimer integrity *in vitro*.

### 426c NFL-C3d fusion trimers elicit higher antibody titers in VRC01-gHL knock-in mice

**Figure 8:**
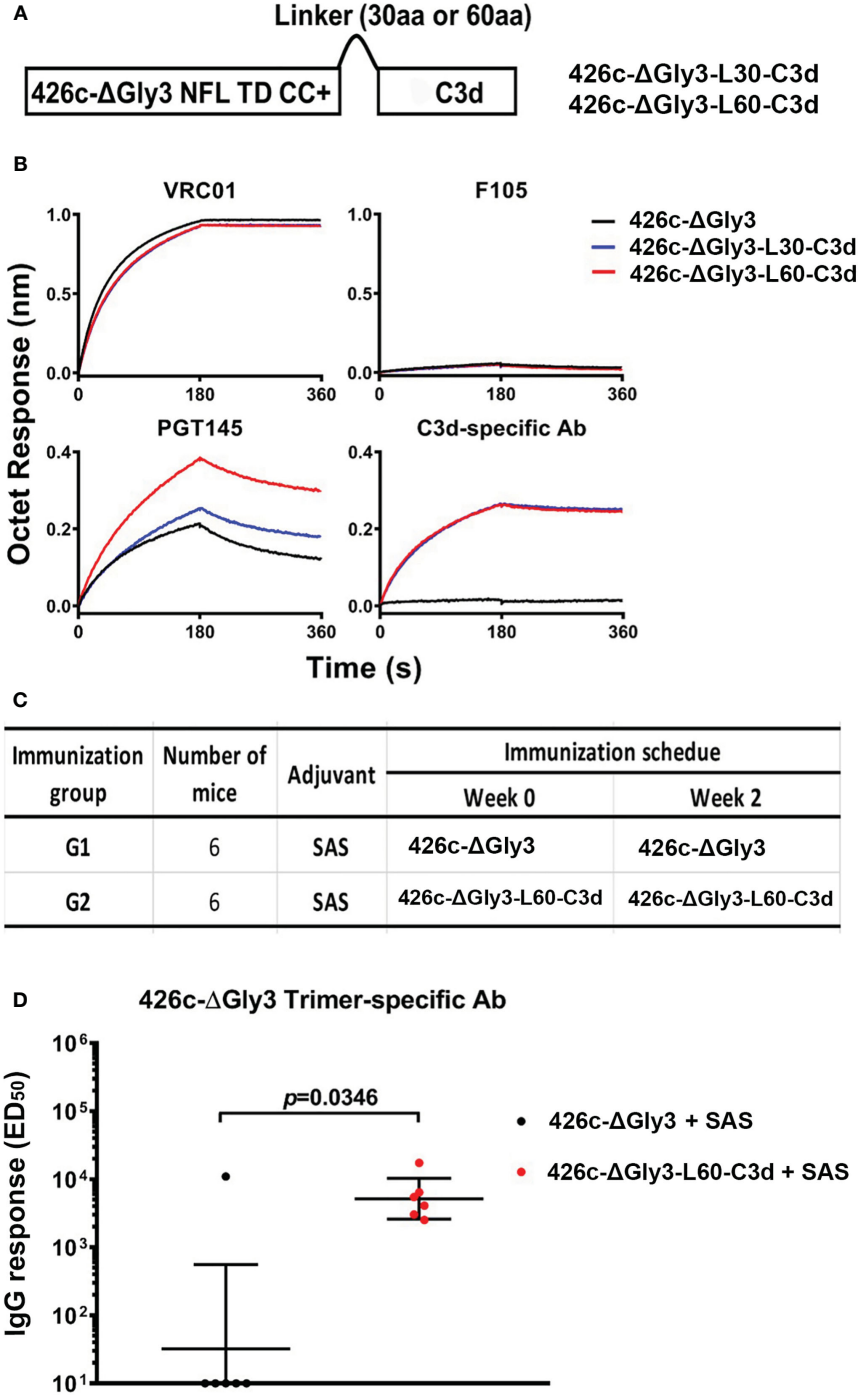
Design, antigenic characterization, and immunogenic analysis of 426c-ΔGly3-C3d trimers. **(A)** Schematic representation of 426c-ΔGly3-C3d fusion trimers. **(B)** 426c-ΔGly3-C3d trimers display favorable antigenic profile against Env-specific bNAbs, non-bNAbs, and mouse C3d-specific Ab. **(C)** Immunization groups and vaccine regimen. **(D)** ED_50_ binding Ab titers with geometric mean and geometric standard derivation against 426c-ΔGly3 trimers (captured by 2G12) in VRC01^gHL^ mice after two immunizations. Data shown are an average of two independent measurements. Pre-immune sera from mice had no detectable specific IgG response and are not shown. Fusion of C3d domains to 426c-ΔGly3 trimers resulted in statistically significant improved binding titers in mice *, indicate P ≤ 0.05; **, indicate P ≤ 0.01; ***, indicate P ≤ 0.001. ns indicates non-significant..

To investigate if we could extend the C3d fusion design to another trimer derived from a different HIV strain, we tested the C3d-fusion design to the 426c Env that is derived from a clade C virus. A stabilized 426c NFL engineered with three N-glycan-deletions (Δ276Δ460Δ463, termed ΔGly3) was used for this purpose (**Figure 8A**), as these trimers have been shown to engage the VRC01 class of precursor antibodies (36). We successfully expressed and purified the 426cΔGly3-C3d fusion trimers possessing both the L30 and L60 linkers (namely, 426cΔGly3-L30-C3d and 426c-ΔGly3-L60-C3d, respectively). Similar antigenic profiles were observed for both the –C3d fusion trimers, compared with their counterpart 426c-ΔGly3 trimers. Moreover, 426c-ΔGly3-L60-C3d trimers showed better recognition by trimer-specific bNAb PGT145 than 426c-ΔGly3-L30-C3d but a similar recognition by VRC01 and C3d-specific Ab (**Figure 8B**). We evaluated the effect of C3d fusion to 426c-ΔGly3 trimers in VRC01^gHL^ mice to assess their capacity to activate antibody responses in this knock-in mouse model (the immunization schedule is shown in **Figure 8C**). After two immunizations in the SAS adjuvant, 426c-ΔGly3-L60-C3d trimers elicited significantly higher Env-specific IgG responses than that elicited by 426c-ΔGly3 trimers (**Figure 8D**). These data indicate that the fusion of C3d improves the ability of Env trimers to activate naïve or germline B cells *in vivo*.

## Discussion

The C3d domain naturally enhances activation of the innate and adaptive arms of immune system by interacting with the CR2 domain of the complement system. The goal of this study was to improve the humoral response to HIV-1 by fusing the molecular adjuvant, C3d, to the “newer generation” cleavage-independent, native-like NFL Env trimers. Accordingly, we generated fusion Env-C3d trimers from the clade B JRFL virus as well as from the clade C 426c virus. Env-C3d achieved a well-ordered trimeric structure and maintained the functional integrity of both the Env and C3d components, respectively. The fusion of C3d did not dramatically affect the antigenicity of the Env trimers; however, it enhanced the ability of the Env trimers to engage and activate B cells *in vitro* and *in vivo*. The SAS adjuvant does not affect the trimer integrity *in vitro*, but it is disruptive to the trimer integrity *in vivo*, resulting in an increased exposure of the V3 epitope. Nonetheless, *in vivo*, there is an additive effect of the SAS adjuvant and the molecular adjuvant C3d to improve the immunogenicity of the Env trimers. The fusion of C3d to the Env trimers improves the elicited antibody responses following vaccination, including Env-specific binding and neutralizing antibodies.

Initially, we screened the linker length between NFL Env and C3d. The 30- residue- (L30) and 60- residue- (L60) long linkers, rather than the 14- residue linker, generate well-ordered Env-C3d fusion trimers. Both Env-C3d variants exhibited similar biochemical, biophysical, structural, and antigenic properties, compared with their counterpart Env trimers. However, the yield of Env-L60-C3d was twofold higher than that of Env-L30-C3d. Env-L60-C3d showed slightly better binding reactivities to bNAbs and activation of B cells *in vitro*, compared with Env-L30-C3d, indicating that the L60 linker is likely better than the L30 linker for the fusion protein to achieve the structural and functional integrity of both the trimer and the C3d components. The 60-residue linker may not be the optimal linker length, as we did not assess intermediate linker lengths using stepwise increases of the G_4_S linker as we previously did to generate the original cleavage-independent NFL Env trimers (5). Nonetheless, the 60-residue linker was efficient to covalently fuse the NFL Env to the molecular adjuvant C3d, to form a well-ordered trimer, and to maintain their respective functional integrity.

Furthermore, the fusion of C3d enhanced the ability of the Env trimers to engage and activate B cells *in vivo*, reflected by the enhanced germinal center formation following two trimer inoculations. These results are consistent with recent work demonstrating that the follicular environment may be lower in proteolytic activity and that targeting to FDCs may benefit the elicited antibody to conformationally sensitive antigens such as the HIV Env trimer (26). In addition, Env-specific binding antibody response, especially in the presence of the SAS adjuvant, was detected with the C3d fusion to the NFL trimer. In the absence of the SAS adjuvant, the enhancement was evident after the first immunization, but not the following boosting, indicating that C3d alone was not sufficient to markedly improve the elicited antibody response. One potential reason is that following repeated immunization, the Env-C3d immunogens elicited *de novo* antibody responses instead of antibody affinity maturation. Furthermore, although mice are genetically inbred and are sufficient to generate Env binding titers, the mouse is not an ideal animal model to elicit neutralizing antibodies against the native HIV-1 Env trimer on the virus. In a previous study, BG505 SOSIP trimers failed to elicit autologous tier 2 NAb response to BG505.T332N in wild-type mice (34); however, more recently, He et al. reported that gp41ECTO-stabilized trimers were able to elicit tier 2 autologous NAbs (37). In this study, the Env-C3d trimers elicited tier 1 neutralizing antibodies only in the presence of the SAS adjuvant.

Elicitation of antibodies against non-neutralizing epitopes on Env may be an “immune distraction” to develop cross- neutralizing antibodies (7). Antibodies elicited against non-broadly neutralizing epitopes comprising the V3 epitope typically neutralize tier 1 MN.3 virus but fail to neutralize tier 2 viruses. A number of strategies have been employed to stabilize HIV-1 Env trimers with reduced exposure of non-neutralizing epitopes, including the V3 epitope (8, 11, 38–41). However, the reduced V3 responses do not enhance the elicitation of autologous or heterologous tier 2 neutralization. In our study, in the absence of the SAS adjuvant, the Env and Env-C3d trimers failed to elicit NAbs against the tier 1 MN.3 virus, which correlates with the low level of V3-specific binding antibody titers. However, in the presence of the SAS adjuvant, there are strong binding antibodies targeting the V3 epitope and neutralizing antibodies against the tier 1 MN.3 virus. The results indicate that, *in vivo*, the Env and Env-C3d trimers maintain a stable trimeric structure when formulated in physical conditions, but the SAS adjuvant is disruptive to the trimer integrity at the V3 epitope.

There have been few studies of how adjuvants affect HIV-1 Env proteins *in vitro* and *in vivo* (13, 42–46). For example, the complete Freund’s adjuvant impairs the conformational integrity of gp120 (45, 46). We previously demonstrated that BG505 NFL or SOSIP trimers retained the quaternary structure more effectively in ISCOMATRIX for up to 7 days than in Adjuplex (42). Adjuplex-induced conformational changes on BG505 NFL can affect the elicitation of tier 2 autologous neutralization and increased tier 1 neutralizing titers (13). Ozorowski et al. recently used a range of analytical techniques to investigate the effects of nine adjuvants on HIV-1 Env SOSIP trimers *in vitro* and found that most of the tested adjuvants have negligible impact on SOSIP trimers, including the SAS adjuvant (43). Similarly, in our study, the SAS adjuvant does not affect the trimers’ integrity *in vitro*, but it seems to affect the integrity of Env trimers *in vivo* by the indirect elicitation of V3-directed mAbs. Therefore, the effect of adjuvants on HIV-1 Env trimers *in vitro* may not always be reflected by their effects on Env trimers *in vivo*. The data indicate that the effects of adjuvant are far more complex and perhaps innate immune activation could also be involved (47, 48). If overall effects conferred by an adjuvant on the meta-stable HIV-1 trimer can only be determined *in vivo*, future selection of the appropriate adjuvant formulation(s) toward an effective HIV-1 vaccine should include both *in vitro* and *in vivo* testing.

In sum, we successfully engineered Env-C3d fusion trimers. The fusion of C3d to the NFL Env trimers improves B- cell antibody responses in animals, and the use of molecular adjuvant C3d could be useful for Env-based vaccines against HIV and other pathogens. The development of HIV-1 Env-C3d fusion immunogens is important as it might circumvent the need for a classical adjuvant, which can adversely affect spike conformation, as these trimers move into the clinic. These results have broader applications for other vaccines that may be extended to conformationally sensitive immunogens derived from other pathogens. Very recently, it was shown that the lymph node follicles may be privileged sites with decreased proteolytic activity that may better maintain native antigen conformation. Thereby, targeting follicular FDCs with Env-C3d fusion trimers may improve the elicitation of antibodies to native-like determinants (26).

Further studies are needed to increase and optimize the number of C3d domains attached per each protomer of the Env trimer. The newer constructs will be tested with different adjuvant formulations that maintain the conformational integrity of the antigens. Immunization studies in animal models other than mice (rabbits, guinea pigs, NHPs for example) with immunoglobulin repertoires that effectively recognize neutralizing determinants on the Env-C3d trimers that can result in tier 2 virus neutralization will provide valuable insights into further efficacy of C3d fusion to Env trimers.

## Materials and methods

### Design of Env-C3d fusion trimer and C3d trimer constructs

To generate Env-C3d fusion trimers, we designed the constructs by fusing mouse C3d (21, 24) to the 3′-end of HIV-1 Env NFL TD CC+. The JRFL NFL TD CC+ (namely, JRFL) (5, 27) and C3d were linked by a flexible linker (G_4_S) of varying lengths (14aa, 30aa, 60aa), namely, JRFL-L14-C3d, JRFL-L30-C3d, and JRFL-L60-C3d, respectively.

426c Env with a three- N-glycan deletion (Δ276 Δ460 Δ463, namely ΔGly3) (36) was used as a template to generate 426c-ΔGly3 NFL TD CC+ (namely, 426c-ΔGly3), as previously described (27). Then, we fused the mouse C3d to the C-terminus of 426c-ΔGly3 through a flexible linker (G4S) of varying lengths (30aa, 60aa), namely, 426c-ΔGly3-L30-C3d and 426c-ΔGly3-L60-C3d, respectively. All native Env signal peptide sequences were replaced by the CD5 leader sequence to increase secretion. The gene constructs were codon optimized for mammalian expression, synthesized (GenScript), and inserted into the CMV/R vector, as previously described (27).

To generate the mouse C3d trimer, we fused the foldon trimeric motif to the C terminus of mouse C3d through two copies of the G_4_S linker. Foldon is a 27-residue trimerization domain at the C-terminal bacteriophage T4 fibritin (49–52). A His-tag was added to the C3d trimer for purification. The fusion gene named C3d-Fd was cloned into an inducible S2 expression vector pMT/BiP/V5-His (Invitrogen), as previously described (53, 54). The amino acid sequence of all the constructs used for this study is shown in **Table S1**.

### Expression and purification of soluble proteins

The constructs expressing JRFL-C3d and 426c-ΔGly3-C3d fusion proteins with different linkers were transiently transfected into suspension 293F cells as previously described (55). Env proteins were harvested 4 days post transfection and purified by lectin affinity chromatography (*Galanthus nivalis*, Vector Labs) followed by size exclusion chromatography (SEC) on a Superdex 200 16/60 or Superdex 200 10/300 GL (GE Healthcare) columns. The trimer peak was subjected to negative selection by the non-neutralizing mAb F105 to remove disordered trimers (31). The flow-through from the F105 column, containing the well-ordered trimers, was resolved by a second SEC step.

C3d-Fd proteins were produced in stably transfected *Drosophila* S2 cells as previously described (53). Briefly, S2 cells were cotransfected with C3d-Fd and selection vector pCoBlast, which contains the blasticidin resistance gene (Invitrogen). After blasticidin selection, stable S2 transfectants were generated that were used for protein production. Briefly, when S2 cells reached 10 M/ml in 1 L of Express Five SFM medium without FBS, 5 μM CdCl_2_ was added into culture medium to induce protein expression; 3 days after the induction, the culture supernatants were harvested, clarified by centrifugation and filtration. Filtered supernatants were purified by Ni-NTA column followed by SEC.

### SDS-PAGE, BN-PAGE, and Western blot analyses

Sodium dodecyl sulfate-polyacrylamide gel electrophoresis (SDS-PAGE), blue-native PAGE (BN-PAGE), and western blot analyses were performed as described elsewhere (55). For western blot analysis, samples were separated through 3%–12% NuPAGE Gels and transferred onto polyvinylidene difluoride membranes (Invitrogen). The membranes were blocked in a solution of Tris-buffered saline containing 5% non-fat dry milk and 0.05% Tween 20 and subsequently probed with anti-Env antibodies (2G12 and VRC01) or biotinylated-goat anti-mouse C3d polyclonal antibody. Proteins were visualized with HRP-conjugated anti-human IgG antibody or streptavidin-peroxidase polymer according to the manufacturer’s instructions (Sigma).

### Differential scanning calorimetry

The thermal transition points (T_m_) of JRFL-C3d and 426c-ΔGly3-C3d fusion trimers were determined by differential scanning calorimetry (DSC) using a MicroCal VP- Capillary instrument (Malvern), as described previously (5, 27).

### Electron microscopy data collection and processing

The purified Env-C3d fusion trimers were analyzed by negative stain electron microscopy (NS-EM). A 3- µl aliquot containing ~0.01 mg/ml of the sample was applied for 15 s onto a carbon-coated 400 Cu-mesh grid that had been glow- discharged at 30 mA for 30 s, then negatively stained with 0.7% uranyl formate for 45 s. Data were collected using an FEI T20 electron microscope operating at 200 kV, with an electron dose of ~45 e^-^/Å^2^ and a magnification of 80,000 × that resulted in a pixel size of 2.74 Å at the specimen plane. Images were acquired with an Eagle 2k × 2k CCD camera (FEI) using a nominal defocus of 1000 nm and the Serial EM software (56). Particles were selected from the micrographs and extracted, and a reference-free 2D class averages were obtained using RELION 2.1.0 (57).

### Binding analysis by biolayer interferometry

Briefly, the BLI analysis was carried out on an Octet Red instrument (ForteBio) with IgGs immobilized on anti-human IgG Fc capture sensors (ForteBio). The Env-C3d trimers were assessed as free analytes in solution (PBS pH 7.4) at a final concentration of 200 nM. Association and dissociation were measured for 180 s. Data were analyzed using ForteBio version 7.1.

For mouse C3d detection, biotinylated-goat anti-mouse C3d polyclonal antibody was immobilized on streptavidin sensors. The fusion Env-C3d or C3d-Fd trimer proteins were assessed as free analytes in solution (PBS pH 7.4) at a final concentration of 200 nM.

For the binding of JRFL-C3d trimers to soluble CR2 protein, his-tagged human CR2 protein (Cat. #10811-H08H, Sino Biological Inc.) was immobilized on anti-HIS sensors. The fusion Env-C3d trimers were assessed as free analytes in solution (PBS pH 7.4) at a final concentration of 200 nM.

### Flowcytometry analysis

To test the capability of Env-C3d fusion trimers to bind CR2 on mouse B cells *in vitro*, we performed flowcytometry analysis. Briefly, we labeled trimers (JRFL, JRFL-L30-C3d, and JRFL-L60-C3d) with Alexa Fluor 680 dye (AF680) according to the manufacturer’s instructions (Thermo Scientific). Then, the PGV04 WEH-I231 cells were treated with 50 ng/μl doxycycline overnight for induction of PGV04 expression on the cell surface. PGV04 expression was confirmed with human constant chain kappa (hCk) expression by flowcytometry analysis. Non-treated PGV04 WEH-I231 cells, with no PGV04 expression on the cell surface, were included as control. WEH-I231 cells with or without PGV04 expression were incubated with AF680- labeled trimers (JRFL, JRFL-L30-C3d, JRFL-L60-C3d) or PBS on ice for 30 min. The cells were then washed twice with wash buffer (PBS containing 1% BSA and 0.02% NaN3) and fixed with 1% formaldehyde in 0.5 ml of wash buffer. Flowcytometry analyses were performed on a BD LSR II flow cytometer (BD Biosciences).

### B- cell activation calcium flux assays

To test the capability of Env-C3d fusion proteins to activate mouse B cells *in vitro*, we performed calcium flux analysis (28, 32). Briefly, PGV04 WEH-I231 cells were treated with 50 ng/μl doxycycline overnight and confirmed for PGV04 expression. PGV04-expressing WEH-I231 cells were suspended at 4 million cells/ml in Hanks balanced salt solution (HBSS), labeled with 0.75 μM Indo-1 (Invitrogen) for 30 min at 37°C, and washed with 2 mM CaCl_2_-HBSS, followed by incubation for 30 min at 37°C. 300 μl of cells at 2 million cells/ml were then stimulated at 37°C with trimers (JRFL, JRFL-L30-C3d, and JRFL-L60-C3d) in solution at a final concentration of 50 μg/ml. Ca^2+^ signals were recorded for 120 s, measuring the 405/485-nm emission ratio of Indo-1 fluorescence upon UV excitation. Calcium flux analysis was performed on an LSR II cytometer (BD Biosciences). Kinetic analysis was performed using FlowJo (Tree Star).

### Animal immunization and sampling

For JRFL-C3d immunization studies, female C57BL/6 (B6) mice (7 to 8 weeks old), six animals per group, were used. B6 mice were inoculated subcutaneously at two sites with 20 μg of soluble trimers in PBS or formulated in 50% v/v Sigma Adjuvant System (SAS, containing 0.5 mg/ml MPLA) in a total volume of 200 μl. Inoculations were done at 0, 4, 10, and 17 weeks. Serum samples were collected 2 weeks before or after each inoculation (see the detailed immunization schedule in **Figure 6A**). One mouse (# ZZ6155) in group 1 died at week 4.

For 426c-ΔGly3-C3d immunization studies, germ-line VRC01 heavy- chain and light- chain (VRC01^gHL^) knock-in mice as described elsewhere (58) were used. Six animals per group were inoculated subcutaneously with 20 μg of soluble 426c-ΔGly3 or 426c-ΔGly3-L60-C3d trimers formulated in SAS in a total volume of 200 μl. Inoculations were done at 0 and 2 weeks. Bleeds were collected before inoculation and 2 weeks after second inoculation. The detailed immunization schedule is shown in **Figure 8C**.

### ELISA analysis

JRFL and 426c trimer-specific IgG-binding titers were determined using 2G12-capture ELISA as previously described (10, 59, 60). Briefly, the 96 half-well ELISA plates were coated with 2G12 mAb (at 2 μg/ml) overnight at 4°C. After blocking and washing, the JRFL trimers at 2 μg/ml were captured onto the plates in blocking buffer for 1 h at 37°C. After washing, the plates were incubated with fivefold serial dilutions of sera starting at 1:20 dilution, for 1 h. The plates were further incubated with horseradish peroxidase (HRP)-coupled anti-mouse IgG at 1:5,000 dilution for 1 h and developed with HRP-3,3′,5,5′-tetramethylbenzidine (TMB) substrate solution. The HRP-TMB reaction was stopped with 0.3 N sulfuric acid, and absorbance was measured at 450 nm. GraphPad Prism software was used to calculate half-maximal effective concentration of serum binding titers (ED_50_).

V3-specific IgG-binding titers were determined by coating 2 μg/ml of JRFL V3 peptide TRPNNNTRKSIHIGPGRAFYTTGEIIGDIRQAH (from amino acids 297–317 according to HxBc2 numbering) on the ELISA plates. The following steps were the same as described above without adding JRFL trimers. Endpoint titers for each sample were calculated based on their OD450 values, and OD450 values over threefold of the background were considered as positive.

To test the effect of the SAS adjuvant on the antigenicity of the Env trimer, we incubated the JRFL trimer containing a His-tag with or without the SAS adjuvant at the same ratio as used in immunization *in vivo* either at room temperature or at 37°C for 24 h. ELISA plates were coated with anti-His antibody. JRFL trimers with or without incubation in adjuvant were captured on the plates, and a panel of monoclonal antibodies were used to detect the integrity of the trimer.

To quantify 426c trimer- specific IgG-binding titers in VRC01^gHL^ knock-in mice sera, we generated mouse VRC01 IgG1 Ab as a standard. Briefly, VRC01 heavy- and light- chain variable regions were cloned with mouse IgG1 Fc or mouse Kappa constant region into pCI-WPRE plasmid (modified pCI plasmid from Promega), respectively. Equal amounts of heavy- and light- chain plasmids were used to transfected 293F cells, and the supernatant was harvested at day 5. VRC01 mouse IgG1 in culture supernatants was purified with a HiTrap Protein G HP column and dialyzed against PBS. For quantification, ELISA plates coated with 2G12 (2 μg/ml) were incubated with the 426c-ΔGly3 trimer at 2 μg/ml. After washing, the plates were incubated with fivefold serially diluted mice sera starting at 1:500 dilution or serially diluted VRC01 mouse IgG1. Purified VRC01 mouse IgG1 was used as standard to calculate the IgG antibody concentration in immunized mouse sera. Non-linear fitting of absorbance data was performed in GraphPad Prism software.

### Germinal center analysis

Germinal center (GC) analysis was performed as previously reported with some adjustments (28). The intramuscular injections were at two sites (100 μl in each site) administered in the thigh muscles of the hind limb of 8-week-old female C57BL/6 mice (eight mice per group) at weeks 0 and 4 with the JRFL trimer (15 μg) or JRFL-L60-C3d trimer (20 μg) formulated in SAS Adjuvant System (Sigma-Aldrich). One group of mice (3 animals) received the SAS adjuvant as a negative control; 2 weeks post second immunization, the draining popliteal and inguinal lymph nodes and spleen were harvested and pressed through a 70-μm cell strainer to obtain a single-cell suspension. The cells were labeled with a LIVE/DEAD viability dye (fixable viability stain, FVS 450, 562247, BD, USA) to exclude dead cells and with PE rat anti-mouse CD19 (BD, 553786), PE Rat Anti-Mouse CD45R/B220 (Clone RA3-6B2, 561878), and fluorescein isothiocyanate (FITC) rat anti-mouse B-cell activation antigen (clone GL7 for germinal center B cells, BD, 553666) markers. Samples were then analyzed on a NovoCyte Flow Cytometer Systems (BD, USA). The post-acquisition analyses, including compensation, were performed using FlowJo software, version 10.8.1 (FlowJo LLC, USA).

### Avidity ELISA assay

Antibody avidity was evaluated by sodium thiocyanate (NaSCN) ELISA as described previously (61) with some modifications. ELISA plates were coated with JRFL Env trimers (at 2 μg/ml) overnight at 4°C. After blocking and washing, serially diluted mice sera were added onto the plates and incubated for 1 h at 37°C before exposure to 1.5 M NaSCN or PBS for 15 min at room temperature. Subsequent steps were performed similarly to 2G12 capture ELISA, as described in the previous section. Endpoint titers for each sample were calculated based on their OD450 values, and OD450 values over threefold of the background were considered as positive. The avidity index was calculated by dividing the endpoint titers for NaSCN-treated samples to those of PBS-treated samples and multiplying by 100.

### Serum neutralization analysis

Serum neutralization analysis was performed in TZM-bl cells, using a single round HIV-1 Env pseudovirus assay (33). To determine the sera dilution that resulted in a 50% reduction in relative luminescence units (RLU), serial dilutions of the sera were performed and the neutralization dose–response curves were fit by non-linear regression using a five-parameter hill slope equation. Diverse HIV-1 virus isolates were used in the neutralization assays. The neutralization titer was expressed as the reciprocal serum dilution that resulted in 50% reduction of RLU (ID_50_).

### Statistical analysis

Analyses were performed with GraphPad Prism v8.0. Different groups in mouse immunizations were compared by two-tailed Mann–Whitney U test. Correlations between the NAb titers and the V3-specific binding antibody titers were assessed by using the non-parametric (two-tailed) Spearman rank correlation method. Data sets were considered statistically significant at a *P* value of ≤ 0.05.

## Data availability statement

The original contributions presented in the study are included in the article/**Supplementary Material**. Further inquiries can be directed to the corresponding author.

## Ethics statement

All mouse studies were performed at TSRI animal facilities under the approval of the TSRI Institutional Animal Care and Use Committee (IACUC) (study numbers - M1708, M1808, M1813, M1905). The committee uses the Guide for Care and Use of Laboratory Animals (National Academy Press), PHS policy, and the AWA as standards. All animal handling protocols were designed to minimize animal discomfort.

## Author contributions

LY, SB, MA, JG, and RTW designed the research studies. LY, SB, RW, TO, MA, EDD, and CAC performed the experiments and data analysis. LY, SB, and RTW wrote the paper. All authors reviewed the results and approved the final version of the manuscript.

## Supplementary material

Supplementary Figure 1 Characterization of JRFL-L14-C3d trimer. **(A)** SEC profile of JRFL-L14-C3d trimer following lectin affinity-purification. Fractions selected for further analysis are labeled. **(B)** Blue-native PAGE (BN-PAGE) analysis of fractions of JRFL-L14-C3d trimers from SEC purification. The band representing the trimer is marked in red. The major peak from SEC purification (fractions 8-10) is comprised of non-specific aggregates with a very small fraction of trimer. **(C)** SEC profile of JRFL NFL trimer following lectin affinity-purification and negative selection by F105.

Supplementary Figure 2 Binding of JRFL-L60-C3d trimer to soluble human CR2 protein. Bio-layer interferometry (BLI) measurements show that JRFL-L60-C3d trimers bind to human CR2 protein. Binding was not observed with the control JRFL trimer lacking the C3d domain.

Supplementary Figure 3 Neutralizing antibody titers correlate with V3-specific binding antibody titers. **(A)** Endpoint V3-specific binding antibody titers as determined by V3-peptide capture ELISA at week 19. **(B)** Midpoint neutralization titers (ID50) against tier 1 MN.3 pseudotyped virus are plotted against the V3-specific binding antibody titers (ED50). The spearman *r*-value and two-tailed *P* value are shown.

Supplementary Figure 4 Effect of SAS adjuvant on the antigenicity of JRFL trimer. ELISA binding curves of selected bNAbs and non-bNAbs to JRFL trimers. The trimers were incubated +/- SAS adjuvant at room temperature (RT) or 37°C for 24 h prior to coating on the ELISA plate.

Supplementary Table 1 Amino acid sequences of all antigens used for this study.
