# Supplementary figures and images for: Fusion of the molecular adjuvant C3d to cleavage-independent native-like HIV-1 Env trimers improves the elicited antibody response
Source: Front Immunol. 2023 May 22;14:1180959. doi: 10.3389/fimmu.2023.1180959 (PMC10239957; doi:10.3389/fimmu.2023.1180959)

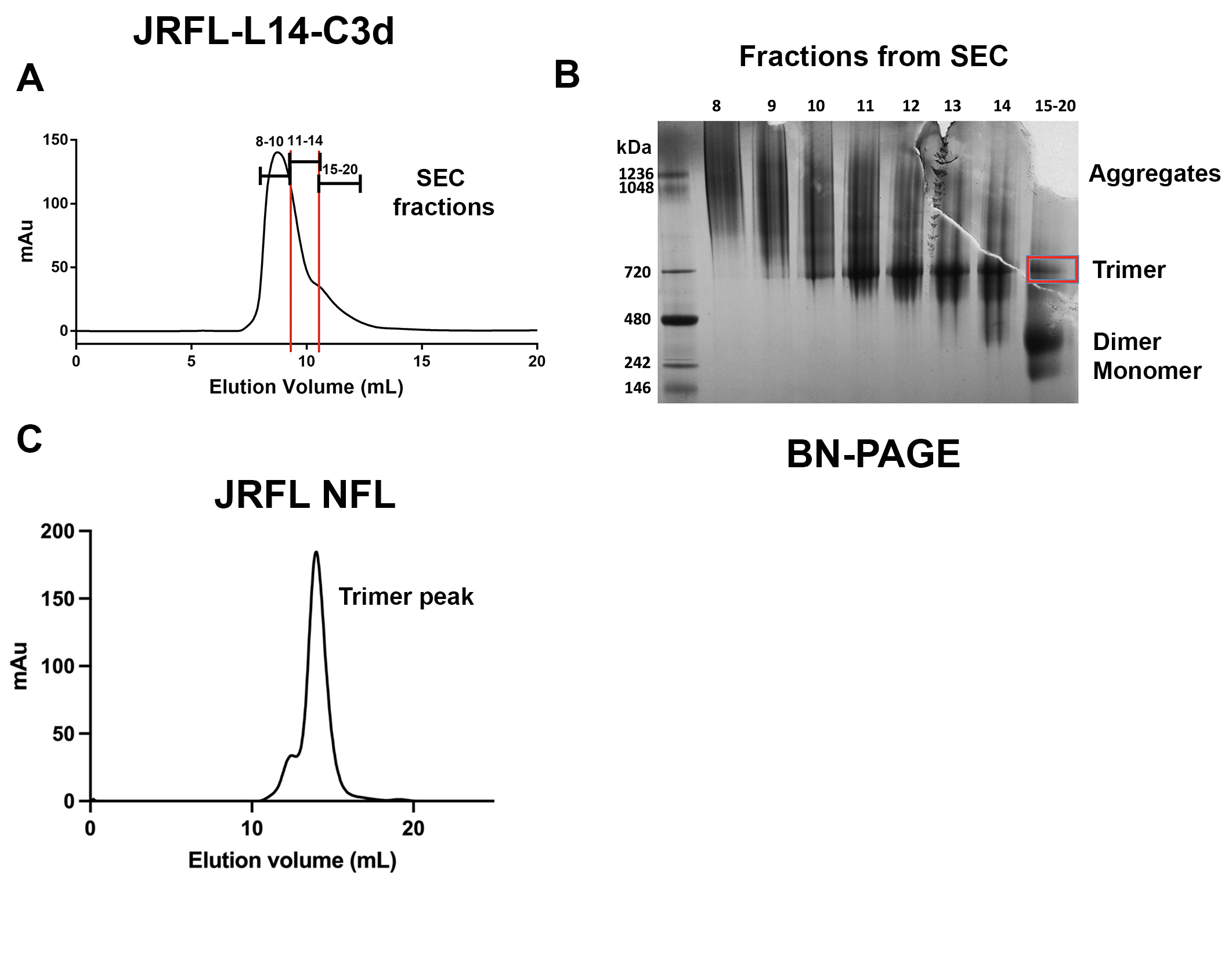

Supplement: Supplementary Figure 1 — Characterization of JRFL-L14-C3d trimer. (A) SEC profile of JRFL-L14-C3d trimer following lectin affinity-purification. Fractions selected for further analysis are labeled. (B) Blue-native PAGE (BN-PAGE) analysis of fractions of JRFL-L14-C3d trimers from SEC purification. The band representing the trimer is marked in red. The major peak from SEC purification (fractions 8-10) is comprised of non-specific aggregates with a very small fraction of trimer. (C) SEC profile of JRFL NFL trimer following lectin affinity-purification and negative selection by F105. [file Image_1.jpeg]

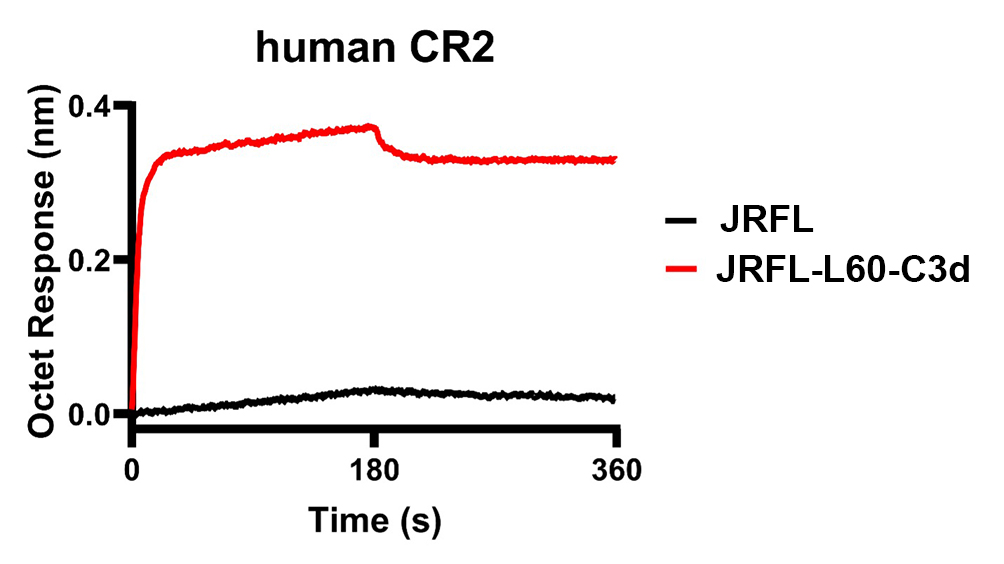

Supplement: Supplementary Figure 2 — Binding of JRFL-L60-C3d trimer to soluble human CR2 protein. Bio-layer interferometry (BLI) measurements show that JRFL-L60-C3d trimers bind to human CR2 protein. Binding was not observed with the control JRFL trimer lacking the C3d domain. [file Image_2.jpeg]

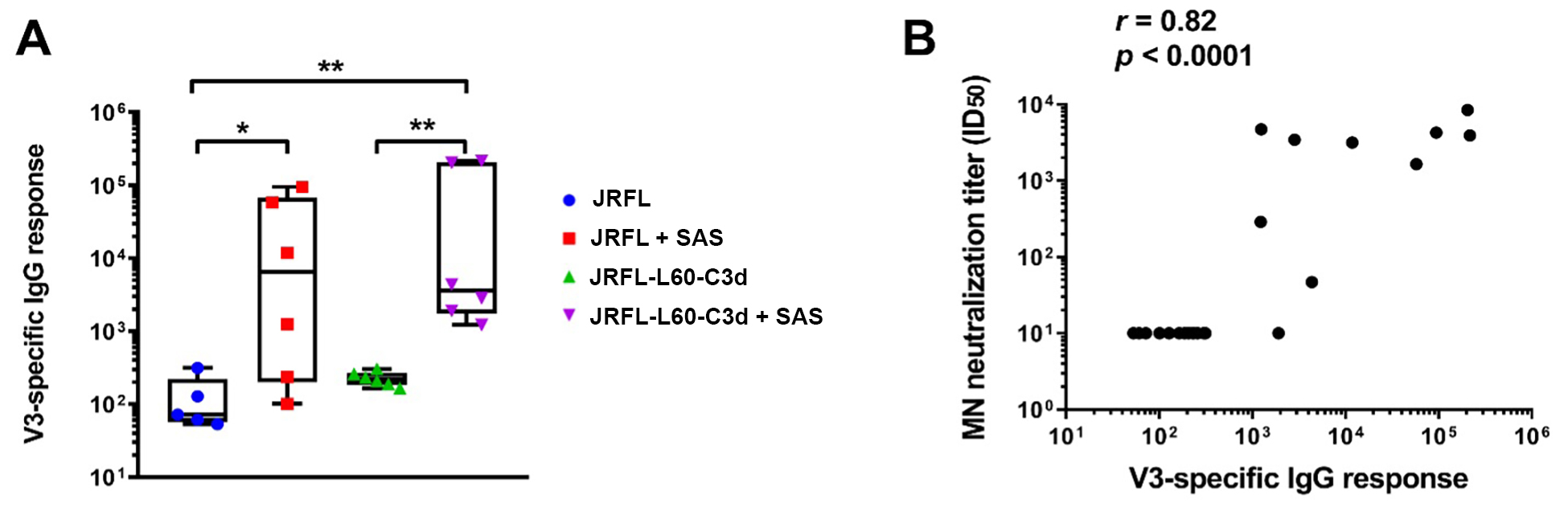

Supplement: Supplementary Figure 3 — Neutralizing antibody titers correlate with V3-specific binding antibody titers. (A) Endpoint V3-specific binding antibody titers as determined by V3-peptide capture ELISA at week 19. (B) Midpoint neutralization titers (ID50) against tier 1 MN.3 pseudotyped virus are plotted against the V3-specific binding antibody titers (ED50). The spearman r-value and two-tailed P value are shown. [file Image_3.jpeg]

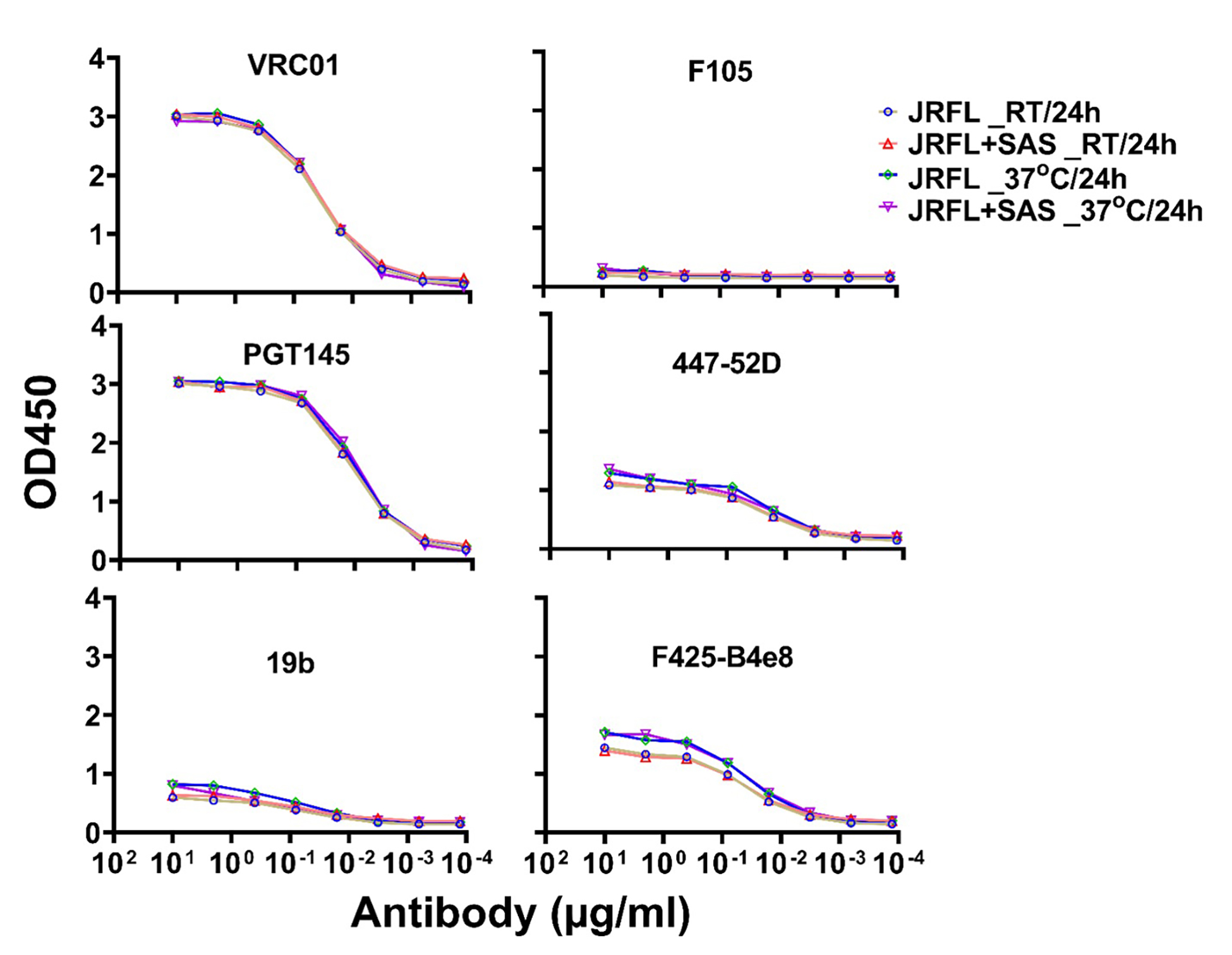

Supplement: Supplementary Figure 4 — Effect of SAS adjuvant on the antigenicity of JRFL trimer. ELISA binding curves of selected bNAbs and non-bNAbs to JRFL trimers. The trimers were incubated +/- SAS adjuvant at room temperature (RT) or 37°C for 24 h prior to coating on the ELISA plate. [file Image_4.jpeg]
