# Supplementary material for: Fusion of the molecular adjuvant C3d to cleavage-independent native-like HIV-1 Env trimers improves the elicited antibody response
Source: Front Immunol. 2023 May 22;14:1180959. doi: 10.3389/fimmu.2023.1180959 (PMC10239957; doi:10.3389/fimmu.2023.1180959)
Supplement: Supplementary Table 1 — Amino acid sequences of all antigens used for this study. [file Table_1.docx]

| **JRFL NFL**  MPMGSLQPLATLYLLGMLVASVLAVEKLWVTVYYGVPVWKDAETTLFCASDAKAYDTEKHNVWATHACVPTDPNPQEVVLENVTEHFNMWKNNMVEQMQTDIISLWDQSLKPCVKLTPLCVTLNCKDVNATNTTNDSEGTMERGEIKNCSFNITTELRDKVQKVYALFYKLDVVPIDNNNTSYRLISCDTSVCTQACPKISFEPIPIHYCAPAGFAILKCNDKTFNGKGPCKNVSTVQCTHGIRPVVSTQLLLNGSLAEEEVVIRSDNFTNNAKTIIVQLKESVEINCTRPNNYTRKSIRIGPGRAFYTTGEIIGDIRQAHCNISRAKWNDTLKQIVIKLREQFENKTIVFNHSSGGDPEIVMHSFNCGGEFFYCNSTQLFNSTWNNNTEGSNNTEGNTITLPCRIKQIINMWQRVGQCMYAPPIRGQIRCSSNITGLLLTRDGGINENGTEIFRPGGGDMRDNWRSELYKYKVVKIEPLGVAPTRAKRRVVQGGGGSGGGGSAVGIGAVRRGFLGAAGSTMGAASMTLTVQARNLLSGIVQQQSNLLRAPEAQQRMLQLGVWGIKQLQARVLAVERYLRDQQLLGIWGCSGKLICTTAVPWNASWSNKSLDRIWNNMTWMEWEREIDNYTSEIYTLIEESQNQQEKNEQELLALD |
| --- |
| **JRFL- L14-C3d**  MPMGSLQPLATLYLLGMLVASVLAVEKLWVTVYYGVPVWKDAETTLFCASDAKAYDTEKHNVWATHACVPTDPNPQEVVLENVTEHFNMWKNNMVEQMQTDIISLWDQSLKPCVKLTPLCVTLNCKDVNATNTTNDSEGTMERGEIKNCSFNITTELRDKVQKVYALFYKLDVVPIDNNNTSYRLISCDTSVCTQACPKISFEPIPIHYCAPAGFAILKCNDKTFNGKGPCKNVSTVQCTHGIRPVVSTQLLLNGSLAEEEVVIRSDNFTNNAKTIIVQLKESVEINCTRPNNYTRKSIRIGPGRAFYTTGEIIGDIRQAHCNISRAKWNDTLKQIVIKLREQFENKTIVFNHSSGGDPEIVMHSFNCGGEFFYCNSTQLFNSTWNNNTEGSNNTEGNTITLPCRIKQIINMWQRVGQCMYAPPIRGQIRCSSNITGLLLTRDGGINENGTEIFRPGGGDMRDNWRSELYKYKVVKIEPLGVAPTRAKRRVVQGGGGSGGGGSAVGIGAVRRGFLGAAGSTMGAASMTLTVQARNLLSGIVQQQSNLLRAPEAQQRMLQLGVWGIKQLQARVLAVERYLRDQQLLGIWGCSGKLICTTAVPWNASWSNKSLDRIWNNMTWMEWEREIDNYTSEIYTLIEESQNQQEKNEQELLALDGSGGGGSGGGGSGSTPAGSGEQHMIGMTPTVIAVHYLDQTEQWGKFGIEKRQEALELIKKGYTQQLAFKQPSSAYAAFNNRPPSTWLTAYVVKVFSLAANLIAIDSHVLCGAVKWLILEKQKPDGVFQEDGPVIHQEMIGGFRNAKEADVSLTAFVLIALQEARDICEGQVNSLPGSINKAGEYIEASYMNLQRPYTVAIAGYALALMNKLEEPYLGKFLNTAKDRNRWEEPDQQLYNVEATSYALLALLLLKDFDSVPPVVRWLNEQRYYGGGYGSTQATFMVFQALAQYQTDVPDHKDLNMDVSFHLPS |
| **JRFL- L30-C3d**  MPMGSLQPLATLYLLGMLVASVLAVEKLWVTVYYGVPVWKDAETTLFCASDAKAYDTEKHNVWATHACVPTDPNPQEVVLENVTEHFNMWKNNMVEQMQTDIISLWDQSLKPCVKLTPLCVTLNCKDVNATNTTNDSEGTMERGEIKNCSFNITTELRDKVQKVYALFYKLDVVPIDNNNTSYRLISCDTSVCTQACPKISFEPIPIHYCAPAGFAILKCNDKTFNGKGPCKNVSTVQCTHGIRPVVSTQLLLNGSLAEEEVVIRSDNFTNNAKTIIVQLKESVEINCTRPNNYTRKSIRIGPGRAFYTTGEIIGDIRQAHCNISRAKWNDTLKQIVIKLREQFENKTIVFNHSSGGDPEIVMHSFNCGGEFFYCNSTQLFNSTWNNNTEGSNNTEGNTITLPCRIKQIINMWQRVGQCMYAPPIRGQIRCSSNITGLLLTRDGGINENGTEIFRPGGGDMRDNWRSELYKYKVVKIEPLGVAPTRAKRRVVQGGGGSGGGGSAVGIGAVRRGFLGAAGSTMGAASMTLTVQARNLLSGIVQQQSNLLRAPEAQQRMLQLGVWGIKQLQARVLAVERYLRDQQLLGIWGCSGKLICTTAVPWNASWSNKSLDRIWNNMTWMEWEREIDNYTSEIYTLIEESQNQQEKNEQELLALDGSGGGGSGGGGSGSGGSGGGGSGGSGGSGSTPAGSGEQHMIGMTPTVIAVHYLDQTEQWGKFGIEKRQEALELIKKGYTQQLAFKQPSSAYAAFNNRPPSTWLTAYVVKVFSLAANLIAIDSHVLCGAVKWLILEKQKPDGVFQEDGPVIHQEMIGGFRNAKEADVSLTAFVLIALQEARDICEGQVNSLPGSINKAGEYIEASYMNLQRPYTVAIAGYALALMNKLEEPYLGKFLNTAKDRNRWEEPDQQLYNVEATSYALLALLLLKDFDSVPPVVRWLNEQRYYGGGYGSTQATFMVFQALAQYQTDVPDHKDLNMDVSFHLPS |
| **JRFL- L60-C3d**  MPMGSLQPLATLYLLGMLVASVLAVEKLWVTVYYGVPVWKDAETTLFCASDAKAYDTEKHNVWATHACVPTDPNPQEVVLENVTEHFNMWKNNMVEQMQTDIISLWDQSLKPCVKLTPLCVTLNCKDVNATNTTNDSEGTMERGEIKNCSFNITTELRDKVQKVYALFYKLDVVPIDNNNTSYRLISCDTSVCTQACPKISFEPIPIHYCAPAGFAILKCNDKTFNGKGPCKNVSTVQCTHGIRPVVSTQLLLNGSLAEEEVVIRSDNFTNNAKTIIVQLKESVEINCTRPNNYTRKSIRIGPGRAFYTTGEIIGDIRQAHCNISRAKWNDTLKQIVIKLREQFENKTIVFNHSSGGDPEIVMHSFNCGGEFFYCNSTQLFNSTWNNNTEGSNNTEGNTITLPCRIKQIINMWQRVGQCMYAPPIRGQIRCSSNITGLLLTRDGGINENGTEIFRPGGGDMRDNWRSELYKYKVVKIEPLGVAPTRAKRRVVQGGGGSGGGGSAVGIGAVRRGFLGAAGSTMGAASMTLTVQARNLLSGIVQQQSNLLRAPEAQQRMLQLGVWGIKQLQARVLAVERYLRDQQLLGIWGCSGKLICTTAVPWNASWSNKSLDRIWNNMTWMEWEREIDNYTSEIYTLIEESQNQQEKNEQELLALDGSGGGGSGGGGSGSGGSGGGGSGGSGGSGSGGGGSGGGGSGSGGGGGGGSGGGGSGGGGSTPAGSGEQHMIGMTPTVIAVHYLDQTEQWGKFGIEKRQEALELIKKGYTQQLAFKQPSSAYAAFNNRPPSTWLTAYVVKVFSLAANLIAIDSHVLCGAVKWLILEKQKPDGVFQEDGPVIHQEMIGGFRNAKEADVSLTAFVLIALQEARDICEGQVNSLPGSINKAGEYIEASYMNLQRPYTVAIAGYALALMNKLEEPYLGKFLNTAKDRNRWEEPDQQLYNVEATSYALLALLLLKDFDSVPPVVRWLNEQRYYGGGYGSTQATFMVFQALAQYQTDVPDHKDLNMDVSFHLPS |
| **426c-ΔGly3**  MPMGSLQPLATLYLLGMLVASVLAVGNLWVTVYYGVPVWKDAETTLFCASDAKAYEKEKHNVWATHACVPTDPNPQEVVLENVTENFNMWKNDMVDQMQTDVISIWDQSLKPCVKLTPLCVTLNCTNVNVTSNSTNVNSSSTDNTTLGEIKNCSFNITTELRDKKQKVYALFYRLDIVPLDNSSNPNSSNTYRLINCNTSTLTQACPKVTFDPIPIHYCAPAGYAILKCNNKTFNGKGPCNNVSTVQCTHGIKPVVSTQLLLNGSLAEEEIVIRSKDLSDNAKIIIVQLNKSVEIVCTRPNNYTRRSIRIGPGQTFYAMGDIIGDIRQAYCNISGRNWSEAVNQVKKKLKEHFPHKNISFQSSSGGDLEITTHSFNCGGEFFYCNTSGLFNDTISNATIMLPCRIKQIINMWQRVGQAIYAPPIKGNITCKSDITGLLLLRDGGDTTDNTEIFRPGGGDMRDNWRSELYKYKVVEIKPLGVAPTRAKRRVVEGGGGSGGGGSAVGIGAVRRGFLGAAGSTMGAASITLTVQARQGLSGIVQQQSNLLRAPEAQQHMLQLGVWGIKQLQTRVLAIERYLKDQQLLGLWGCSGKLICTTAVPWNISWSNKSKEEIWENMTWMQWDREIGNYTNTIYRLLEESQNQQENNEKDLLALD |
| **426c-ΔGly3- L30-C3d**  MPMGSLQPLATLYLLGMLVASVLAVGNLWVTVYYGVPVWKDAETTLFCASDAKAYEKEKHNVWATHACVPTDPNPQEVVLENVTENFNMWKNDMVDQMQTDVISIWDQSLKPCVKLTPLCVTLNCTNVNVTSNSTNVNSSSTDNTTLGEIKNCSFNITTELRDKKQKVYALFYRLDIVPLDNSSNPNSSNTYRLINCNTSTLTQACPKVTFDPIPIHYCAPAGYAILKCNNKTFNGKGPCNNVSTVQCTHGIKPVVSTQLLLNGSLAEEEIVIRSKDLSDNAKIIIVQLNKSVEIVCTRPNNYTRRSIRIGPGQTFYAMGDIIGDIRQAYCNISGRNWSEAVNQVKKKLKEHFPHKNISFQSSSGGDLEITTHSFNCGGEFFYCNTSGLFNDTISNATIMLPCRIKQIINMWQRVGQAIYAPPIKGNITCKSDITGLLLLRDGGDTTDNTEIFRPGGGDMRDNWRSELYKYKVVEIKPLGVAPTRAKRRVVEGGGGSGGGGSAVGIGAVRRGFLGAAGSTMGAASITLTVQARQGLSGIVQQQSNLLRAPEAQQHMLQLGVWGIKQLQTRVLAIERYLKDQQLLGLWGCSGKLICTTAVPWNISWSNKSKEEIWENMTWMQWDREIGNYTNTIYRLLEESQNQQENNEKDLLALDGSGGGGSGGGGSGSGGSGGGGSGGSGGSGSTPAGSGEQHMIGMTPTVIAVHYLDQTEQWGKFGIEKRQEALELIKKGYTQQLAFKQPSSAYAAFNNRPPSTWLTAYVVKVFSLAANLIAIDSHVLCGAVKWLILEKQKPDGVFQEDGPVIHQEMIGGFRNAKEADVSLTAFVLIALQEARDICEGQVNSLPGSINKAGEYIEASYMNLQRPYTVAIAGYALALMNKLEEPYLGKFLNTAKDRNRWEEPDQQLYNVEATSYALLALLLLKDFDSVPPVVRWLNEQRYYGGGYGSTQATFMVFQALAQYQTDVPDHKDLNMDVSFHLPS |
| **426c-Δgly3- L60-C3d**  MPMGSLQPLATLYLLGMLVASVLAVGNLWVTVYYGVPVWKDAETTLFCASDAKAYEKEKHNVWATHACVPTDPNPQEVVLENVTENFNMWKNDMVDQMQTDVISIWDQSLKPCVKLTPLCVTLNCTNVNVTSNSTNVNSSSTDNTTLGEIKNCSFNITTELRDKKQKVYALFYRLDIVPLDNSSNPNSSNTYRLINCNTSTLTQACPKVTFDPIPIHYCAPAGYAILKCNNKTFNGKGPCNNVSTVQCTHGIKPVVSTQLLLNGSLAEEEIVIRSKDLSDNAKIIIVQLNKSVEIVCTRPNNYTRRSIRIGPGQTFYAMGDIIGDIRQAYCNISGRNWSEAVNQVKKKLKEHFPHKNISFQSSSGGDLEITTHSFNCGGEFFYCNTSGLFNDTISNATIMLPCRIKQIINMWQRVGQAIYAPPIKGNITCKSDITGLLLLRDGGDTTDNTEIFRPGGGDMRDNWRSELYKYKVVEIKPLGVAPTRAKRRVVEGGGGSGGGGSAVGIGAVRRGFLGAAGSTMGAASITLTVQARQGLSGIVQQQSNLLRAPEAQQHMLQLGVWGIKQLQTRVLAIERYLKDQQLLGLWGCSGKLICTTAVPWNISWSNKSKEEIWENMTWMQWDREIGNYTNTIYRLLEESQNQQENNEKDLLALDGSGGGGSGGGGSGSGGSGGGGSGGSGGSGSGGGGSGGGGSGSGGGGGGGSGGGGSGGGGSTPAGSGEQHMIGMTPTVIAVHYLDQTEQWGKFGIEKRQEALELIKKGYTQQLAFKQPSSAYAAFNNRPPSTWLTAYVVKVFSLAANLIAIDSHVLCGAVKWLILEKQKPDGVFQEDGPVIHQEMIGGFRNAKEADVSLTAFVLIALQEARDICEGQVNSLPGSINKAGEYIEASYMNLQRPYTVAIAGYALALMNKLEEPYLGKFLNTAKDRNRWEEPDQQLYNVEATSYALLALLLLKDFDSVPPVVRWLNEQRYYGGGYGSTQATFMVFQALAQYQTDVPDHKDLNMDVSFHLPS |
